# Fifteen cases clinical analysis of wedge-shaped resection of uterus treating adenomyosis—CONSORT

**DOI:** 10.1097/MD.0000000000003805

**Published:** 2016-06-17

**Authors:** ShanRong Shu, Xin Luo, ZhiXin Wang, YuHong Yao

**Affiliations:** Department of Obstetrics and Gynecology, The First Affiliated Hospital of JiNan University, Guangzhou, People's Republic of China.

**Keywords:** adenomyosis, dysmenorrhea, menorrhagia, wedge-shaped

## Abstract

To investigate the improvement of dysmenorrhea and menorrhagia after wedge-shaped resection of uterus. The clinical data of 15 patients who experienced wedge-shaped resection of uterus for adenomyosis were retrospectively analyzed from September 2012 to October 2013. We use the amount of the completed soaked napkins to measure the menstrual blood volume, and the visual analog scale to evaluate the degree of dysmenorrhea. We used the 2 index to evaluate the improvement of dysmenorrhea and menorrhagia after operation. All operations were successful, no serious complication occurred. Before the operation, all 15 patients used more than 25 pieces of completed soaked napkins, after the operation, 13 patients had significantly decreased menstrual flow, the average amount of completed soaked napkins was 3.6. Meanwhile, 2 patients had no menstrual after surgery. Before the operation, among the 10 patients with severe dysmenorrhea, 9 patients had significant relief on pain, they only experienced slight pain after surgery, only 1 patient still experienced moderate pain. Two patients with slight pain had no pain after operation. Among the 3 patients with moderate pain, 2 patients experienced slight pain and 1 patient felt no pain after operation. The wedge-shaped resection of uterus is a safe and effective procedure to significantly reduce menorrhagia and alleviate the extent of dysmenorrhea, which is a promising alternative for patient who suffered from dysmenorrhea and menorrhagia for adenomyosis.

## 1 Introduction

Adenomyosis is common in women of childbearing age. The signs and symptoms include progressive dysmenorrhea, menorrhagia, abnormal uterine bleeding, enlarged uterus, dyspareunia, and infertility, which can seriously affect the patient's quality of life. Currently, the treatments mainly include pharmacotherapy, such as oral contraceptives, nonsteroidal antiinflammatory painkillers, progesterone, gonadotropin-releasing hormone analog (GnRHa) and surgery.^[1]^

Drug treatment can relieve the symptom in short-term, but drug withdrawal usually results in the recurrence. Although adenomyosis mainly occurs in multiparous women, those women still want to preserve uterine. What's more, the newly recognized theory about endometriosis is eutopic endometrium determinism.^[2]^ So how to clear away eutopic endometrium which caused dysmenorrhea and menorrhagia but preserve uterus and ovary function become the focus of many gynecologist, especially for those patients who refused to take medicine, but had severe dysmenorrhea and heavy menstruation. Wedge-shaped resection of uterus is the procedure in which the uterus body and most endometrium are deleted, and the remnant uterus muscle is sutured. The advantage of this procedure is significant relieve patients’ discomfort and reserving uterine artery ascending branch, which can delay the occurrence of menopause symptoms.

The aim of the present study is to analyze the improvement of symptoms after the wedge-shaped resection of uterus, and to explore whether wedge-shaped resection of uterus is a promising treatment for adenomyosis inducing dysmenorrhea and menorrhagia.

## 2 Subjects and methods

### 2.1 Patients

Fifteen patients were admitted in our study during September 2012 to October 2013. This study was approved by the institutional ethics committee, and informed consent was taken from all subjects. The including criteria were: the patients were multiparous and had no desire for giving birth to another child, there were no other reasons leading to dysmenorrhea and menorrhagia and none of the patients had any history of serious health conditions. All of them underwent wedge-shaped resection of uterus, and pathological examination verified adenomyosis. The age of patients ranged from 32 to 42 years old, the mean age was 33.2 ± 3.2 years old. All the patients were multiparous women, 10 patients gave birth to 2 children. Six patients had minor uterus myoma protruding serosal layer, the diameter of which was 2 cm. Two patients had ovary endometriosis which was discovered during the operation. After surgery, all the patients were followed up for 7 to 12 months. No patient had recurrent lesion.

### 2.2 Surgery method

**Figure 1:**
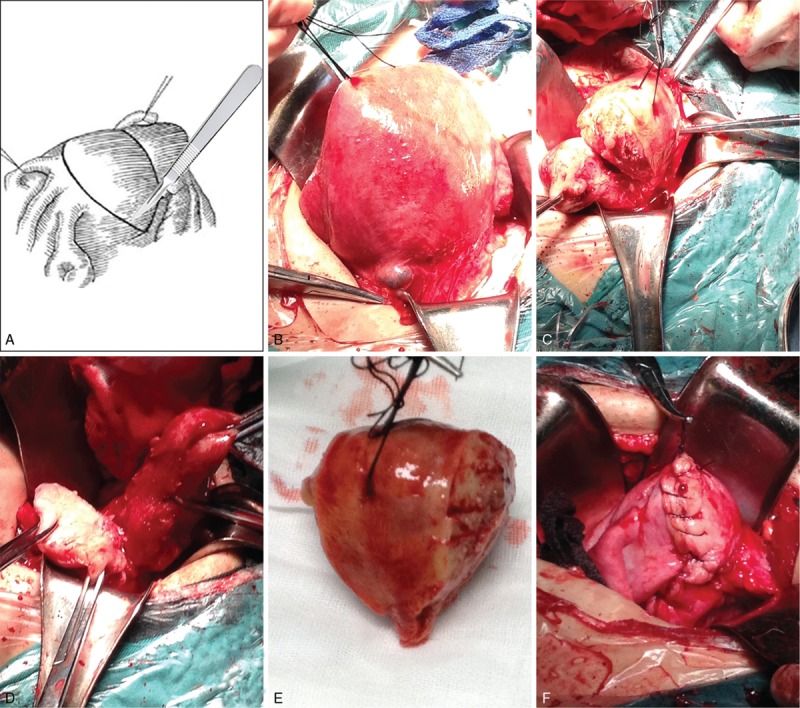
To explain how to perform the wedge-shaped resection of uterus. (A) The schematic diagram of the main body of uterus and most endometrium resected from anterior and posterior wall of uterus. (B) Before the operation, the uterus was enlarged. (C) The main body of uterus and most endometrium was resected. (D) The remaining uterus. (E) The resected main body of uterus. (F) The remaining uterus was sutured.

The uterus was pulled outside of the abdominal incision. This technique involves determining the location and border of lesions by inspection and/or palpation. For those ill-defined borders, we resected the most uterus body as much as possible, so the width of left uterus in each side was 2 cm. Longitudinal incision of the uterus wall along the borders of adenomyoma. Sharp and blunt separation of the lesion with scissors to remove the whole adenomyoma and part of normal myometrium. Thus, we resected the main body and most endometrium from anterior and posterior wall of the uterus (Fig. 1), which appeared an inverted triangle, the vertex was the endocervix, the baseline was the uterus fundus. Then we separately sutured the anterior and posterior wall of the uterus with 1–0 absorbable thread continuously, in the fundus of uterus, the sutured thread was knotted respectively, which could avoid the fracture of one thread leading to the rupture of the whole uterus and prevent the spontaneous rupture of uterus. In some cases, we firstly sutured the endometrial cavity with 2–0 absorbable thread and suture 2 or more layers of the seromuscular layer if the myometrium was very thick, which could strengthen the suture and avoid the formation of cavity. In some patients, we also separated the pelvic adhesion, stripped ovary cyst, and excised minor myoma.

### 2.3 Outcome measurements

The degree of dysmenorrheal (pain) was assessed using the visual analog scale. The left end of the scale was set at 0 (no pain) and the right end of scale at 100 (intolerable pain). The scale of 10 to 30 was slight pain, 40 to 60 was moderate pain, 70 to 100 was severe pain. The degree of pain was self-reported by patients. For menstrual flow, the number of sanitary napkins used 1 day, which was completed soaked was counted. When the amount of used napkin was more than 25 pieces, we determined menorrhagia.

### 2.4 Follow-up

We called the patients to ask for the situation of dysmenorrheal relief and menstrual flow every month after operation, also, we advised the patient to take B ultrasonic examination every 3 months after operation to find the recurrence.

### 2.5 Statistical analysis

The SPSS 13.0 software package was used for statistical analysis. *P* < 0.05 was considered statistically significant.

## 3 Results

### 3.1 Operation situation

All the operations were successful, no serious complication occurred. The lesion of adenomyosis ranged from 3 cm × 2 cm to 10 cm × 8 cm. At the same time, pelvic adhesion lysis was taken in 6 patients, ovary chocolate cyst was stripped in 2 patients, and minor myoma was excised in 6 patients.

### 3.2 The improvement of symptoms

According to the visual analog scale, when the score was <30, we considered the pain was slight, when the score ranged from 40 to 60, the pain was moderate, when the score was above 70, the pain was sever. In our study, before the operation, 10 patients had severe dysmenorrheal, 3 patients had moderate dysmenorrhea, and 2 patients had slight dysmenorrhea. After operation, 3 patients undergoing moderate pain had mild pain, the remission rate was as high as 100%, especially, 1 patient felt no pain after operation. In the 10 patients who suffered from severe dysmenorrheal, only 1 patient still experienced moderate pain. All patients suffered from menorrhagia. The used napkins was counted, as shown in Table 1, 15 patients all used a great many napkins, which was as high as 50 pieces in 1 day. After operation, the menstruation flow was significantly decreased; meanwhile, 2 patients had no menstrual flow after operation.

**Table 1:**
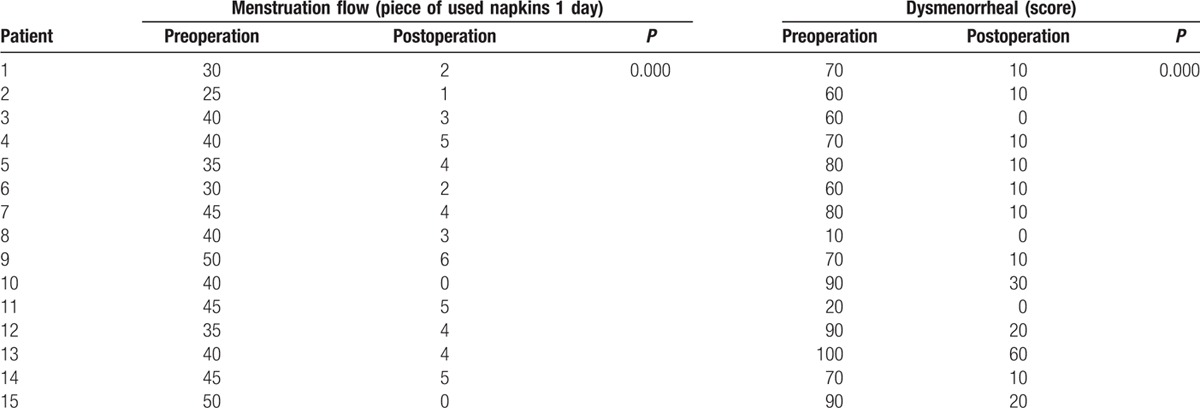
The menstruation flow and dysmenorrheal of 15 patients before and after operation.

### 3.3 B ultrasonic examination

**Figure 2:**
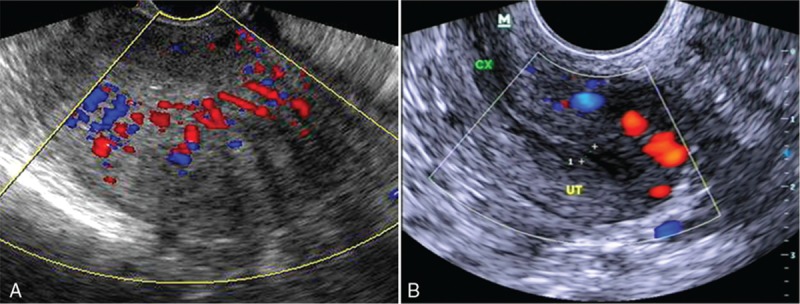
The patients with adenomyosis took B ultrasonic examination before or after the operation. Before the operation, we found enlarged uterus with rich blood flow signal. After the operation, the uterus was shrunk. (A) Before the operation, the uterus was enlarged with rich blood flow signals. (B) After the operation, the uterus was shrunk with poor blood flow signals.

The adenomyosis showed low density mass in the enlarged uterus with or without blood flow signal. In our study, B ultrasonic taken 3 months later after operation showed shrunk uterus with little or no blood flow signal as shown in Fig. 2.

## 4 Discussion

Adenomyosis is a common benign gynecological disorder affecting premenopausal women. It is characterized by the presence of ectopic endometrial glands and stroma in myometrium and hyperplasia of adjacent smooth muscle.^[3]^ The symptoms of adenomyosis include dysmenorrhea, menorrhagia, abnormal uterine bleeding, and diffuse uterine enlargement, sometimes leading to pelvic pressure and frequent urination. Hysterectomy, which eliminate the underlying source of symptoms, is the most common treatment for adenomyosis.^[4]^ For women who wish to preserve their uterus, the alternative options include hormonal therapy,^[5,6]^ endometrial ablation therapy,^[7]^ and uterine artery embolization.^[8,9]^ Hormonal treatment offer only temporary alleviation of symptoms and is associated with some severe side effects,^[10]^ endometrial ablation is an alternative when the depth of myometrial penetration is limited,^[7]^ uterine artery embolization has elicited severe complication.^[8,9]^

Wedge-shaped resection of uterus removed uterus body and most endometrium, which disposed lesions but preserved the ascending uterus artery, so the function of ovary was not affected. This was the advantage of the procedure for it delayed the occurrence of menopausal syndrome, but the weakness of this procedure was the loss of the productive function, so it was only suitable for multiparous women who had no desire for another child.

Wedge-shaped resection of uterus could significantly relieve the symptom of dysmenorrhea and menorrhagia. As shown in the period of follow up, 2 patients had no menstruation and menstruation flow was significantly decreased for the deletion of endometrium. Also, patients who had high menstrual pain score before treatment were free of menstrual pain after surgery. They reported a significant improvement in their quality of life. All the patients had no adverse effect or complication; recurrences were not recorded during the treatment or follow-up period.

Our study suggests that clinical improvement in symptomatic adenomyosis is achievable with the wedge-shaped resection of uterus, which may be a promising alternative for patients who suffered from dysmenorrhea and menorrhagia for adenomyosis but still want to preserve the uterus.
